# Current and emerging therapeutic strategies for preventing inflammation and aggrecanase-mediated cartilage destruction in arthritis

**DOI:** 10.1186/s13075-014-0429-9

**Published:** 2014-09-26

**Authors:** Carolyn M Dancevic, Daniel R McCulloch

**Affiliations:** School of Medicine and Molecular and Medical Research SRC, Faculty of Health, Deakin University, 75 Pigdons Road, Waurn Ponds, VIC 3216 Australia

## Abstract

Arthritis is a multifactorial disease for which current therapeutic intervention with high efficacy remains challenging. Arthritis predominately affects articular joints, and cartilage deterioration and inflammation are key characteristics. Current therapeutics targeting inflammatory responses often cause severe side effects in patients because of the systemic inhibition of cytokines or other global immunosuppressive activities. Furthermore, a lack of primary response or failure to sustain a response to treatment through acquired drug resistance is an ongoing concern. Nevertheless, treatments such as disease-modifying anti-rheumatic drugs, biological agents, and corticosteroids have revealed promising outcomes by decreasing pain and inflammation in patients and in some cases reducing radiographic progression of the disease. Emerging and anecdotal therapeutics with anti-inflammatory activity, alongside specific inhibitors of the A Disintegrin-like And Metalloproteinase domain with Thrombospondin-1 repeats (ADAMTS) cartilage-degrading aggrecanases, provide promising additions to current arthritis treatment strategies. Thus, it is paramount that treatment strategies be optimized to increase efficacy, reduce debilitating side effects, and improve the quality of life of patients with arthritis. Here, we review the current strategies that attempt to slow or halt the progression of osteoarthritis and rheumatoid arthritis, providing an up-to-date summary of pharmaceutical treatment strategies and side effects. Importantly, we highlight their potential to indirectly regulate ADAMTS aggrecanase activity through their targeting of inflammatory mediators, thus providing insight into a mechanism by which they might inhibit cartilage destruction to slow or halt radiographic progression of the disease. We also contrast these with anecdotal or experimental administration of statins that could equally regulate ADAMTS aggrecanase activity and are available to arthritis sufferers worldwide. Finally, we review the current literature regarding the development of synthetic inhibitors directed toward the aggrecanases ADAMTS4 and ADAMTS5, a strategy that might directly inhibit cartilage destruction and restore joint function in both rheumatoid arthritis and osteoarthritis.

## Arthritis

Arthritis is a debilitating degenerative disease of articular joints and is characterized predominately by articular cartilage degradation, alterations to subchondral bone mass, and localized inflammation. The substantial impact on health-care budgets in Western nations is evidenced by an estimated health-care burden of 50 million adults (22%, or approximately 1 in 5) in the US and, worldwide, an estimated 175 million adults have some form of arthritic disease [1,2]. Inflammatory cytokines such as IL-1, IL-6, and TNF-α expressed locally in the articular joint cause inflammation, stimulating the production of cartilage-degrading zinc-dependent matrix metalloproteinases (MMPs) such as MMP-1, MMP-2, MMP-3, MMP-9, and MMP-13 and the A Disintegrin-like And Metalloproteinase domain with Thrombospondin-1 repeats (ADAMTS) enzymes, predominately ADAMTS4 and ADAMTS5 or the ‘aggrecanases’ [3,4].

### Roles of matrix metalloproteinases and ADAMTS in cartilage formation

An equilibrium exists between metalloproteinases and their inhibitors to maintain a balance between anabolism and catabolism in articular cartilage. In arthritis, disequilibrium favors the catabolism of cartilage whereby protease activity outweighs their inhibition by tissue inhibitors of metalloproteinases (TIMPs). Although MMP and ADAMTS enzymes are responsible for the degradation of cartilage in arthritic disease, their roles in cartilage development and remodeling are crucial for joint formation and homeostasis. MMP-1 and −2 are localized in synovium and joint articular surfaces in human fetal limbs at 7 to 14 weeks’ gestation, suggesting roles for these proteases in the development and remodeling of synovial tissue and articular cartilage [5]. Studies using homozygous *Mmp-9*-null mice revealed its requirement for angiogenesis and ossification of the developing growth plate since these mice exhibited delayed apoptosis, ossification, and vascularization of hypertrophic chondrocytes, resulting in progressive growth-plate lengthening [6]. Furthermore, *Mmp-13*-null mice exhibit defects in growth-plate cartilage with expanded hypertrophic chondrocyte zones and increased trabecular bone as well as increased interstitial collagen accumulation, with combinatorial *Mmp-9* and *Mmp-13* knockout mice displaying an exacerbated phenotype, suggesting synergy between these two proteases in cartilage and bone formation [7,8]. Importantly, mutations in *MMP-9* and *MMP-13* in humans cause genetic disorders in bone and cartilage growth and developmental phenotypes such as metaphyseal dysplasia and spondyloepimetaphyseal dysplasia, Missouri type [9,10], which are disorders of abnormal growth and development of long bones and vertebrae. *Mmp-14* (MT1-MMP)-deficient mice display severe skeletal abnormalities, including impaired vascularization of epiphyseal cartilage, leading to delayed ossification and hypertrophic zone lengthening, revealing a role for *Mmp-14* in angiogenesis and bone growth [11]. Significantly, human mutations in *MT1-MMP* cause Winchester syndrome, which is associated with progressive osteolysis, osteoporosis, and joint erosions [12]. It has not yet been established whether ADAMTS4 or ADAMTS5 has a role in the development and growth of cartilage and bone, although their expression is upregulated in arthritic disease. Other ‘aggrecanases’ include ADAMTS1, ADAMTS9, and ADAMTS15, which may have roles during cartilage and bone development. Although *Adamts1* mRNA is expressed in growth-plate and articular cartilage during normal mouse development and is upregulated in hypertrophic differentiation of growth-plate chondrocytes, it does not play a significant role in cartilage and bone development and growth [13] or in arthritis. *Adamts9* mRNA is also expressed from 13.5 days post-coitus during mouse embryogenesis in the perichondrium, the proliferative zone in the growth plate and bone [14], but roles for ADAMTS9 have not yet been elucidated in cartilage and bone development or in arthritic disease. Furthermore, ADAMTS15 is expressed in chondrocytes and perichondrium of the synovial joints in the developing mouse embryo at 15.5 days post-coitus; however, its function in the joint during development or arthritis has not yet been elucidated [15]. Aggrecan degradation facilitated by MMP and ADAMTS enzymes is a process that occurs within normal and arthritic cartilage, signifying a role for these proteases in normal turnover as well as in arthritis [16], whereas structural changes in aggrecan occur during healthy aging [17].

### Enzymatic processing of joint cartilage

MMP activity is upregulated in arthritic cartilage and synovial fluid [18,19], which correlates with type II collagen cleavage [20]. The collagenases (MMP-1, MMP-8, and MMP-13) preferentially degrade type II collagen (collagen II) at Gly^775^↓^776^Leu causing loss of its trimeric structure, exposing it to further degradation [21]. MMP-2 and MMP-9 (the gelatinases) and MMP-3 (stromelysin), which degrade non-collagen matrix components of the joint, also promote further degradation of denatured collagen II after cleavage by collagenases [22]. MMPs also degrade aggrecan; MMP-3, first isolated from human articular cartilage, cleaves the Asn^341^↓^342^Phe bond of aggrecan in its interglobular domain (IGD) [23,24]. However, it was recently shown that MMP-generated aggrecan fragments are involved predominately in normal aggrecan turnover and that their preferred cleavage site is located C-terminal to the IGD and that these fragments may have a lesser role in aggrecan degradation in knee injuries and osteoarthritis (OA) in human cartilage [25].

**Figure 1:**
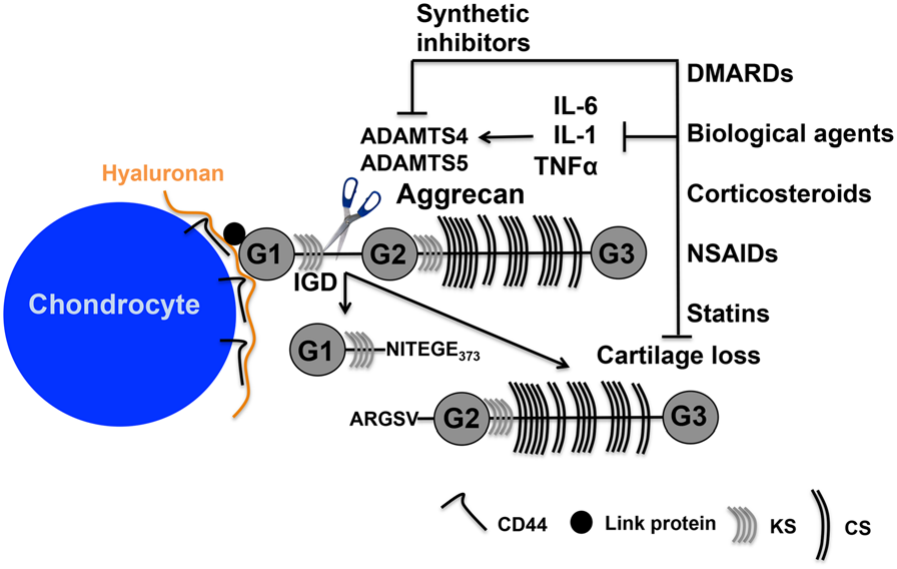
**The destruction of aggrecan and therapies that slow radiographic progression of arthritis.** Full-length aggrecan resides in the pericellular matrix attached to hyaluronan via link protein. Hyaluronan is bound to its cell surface receptor CD44 on articular chondrocytes. ADAMTS4 and ADAMTS5 cleavage (scissors) within the interglobular domain (IGD) of aggrecan is the most detrimental to cartilage function in arthritis as it releases the entire chondroitin sulphate (CS)-modified C-terminus into the synovium. Inhibitors of cytokine activity or ADAMTS5 may prevent cartilage loss directly. ADAMTS, A Disintegrin-like And Metalloproteinase domain with Thrombospondin-1 repeats; DMARD, disease-modifying anti-rheumatic drug; G, immunoglobulin-like domain; IL, interleukin; KS, keratan sulphate; NSAID, non-steroidal anti-inflammatory drug; TNF-α, tumor necrosis factor-alpha.

In contrast to collagen II degradation by MMPs, aggrecan degradation by aggrecanases is an early and reversible event [26]. Furthermore, since aggrecan prevents collagen II degradation and therefore may impart overall cartilage protection [27,28], inhibiting aggrecan degradation via the ADAMTS aggrecanases may be a crucial therapeutic strategy to prevent further collagen II degradation. ADAMTS4 and ADAMTS5/ADAMTS11 were first described in 1999 [29-31] and cleave aggrecan in its C-terminal chondroitin sulphate (CS)-rich domains (Figure 1) at the following sites: SELE^1545^↓^1546^GRGT, KEEE^1714^↓^1715^GLGS, TAQE^1819^↓^1820^AGEG, and ISQE^1919^↓^1920^LGQR; however, the most detrimental cleavage is thought to occur within its IGD at TEGE^373^↓^374^ARGS (Glu^373^↓^374^Ala bond), generating G1-NITEGE fragments that release the entire CS-rich region into the synovial fluid compromising joint function (reviewed in Fosang and colleagues [32]) (Figure 1).

### Aggrecanases and their contribution to arthritis

In 2005, two independent landmark studies demonstrated that ADAMTS5 catalytic inactivation protected mice from experimentally induced OA and rheumatoid arthritis (RA) [33,34] but that ADAMTS4-deficient or catalytically inactivated mice did not show this same protection. However, whether ADAMTS4 or ADAMTS5 is predominately responsible for the cleavage of aggrecan in arthritis in humans remains controversial. Although ADAMTS5 cleaves aggrecan extensively in human arthritic synovium and is abundant and widely distributed in human OA cartilage [35], other data have indicated that both ADAMTS4 and ADAMTS5 cooperate to mediate aggrecan degradation in human articular cartilage explants [36].

Since arthritis is a disease of the entire joint, ADAMTS4 and ADAMTS5 may have variable activity depending on their localization and which cytokines are present to stimulate their gene expression and activation profile. IL-1α and TNF-α induction of *Adamts5* was found to occur predominately in synovium and the patellar but not in femoral head or tibial joint cartilage in *ex vivo* mouse joints, indicating that ADAMTS5 may not be the predominant aggrecanase in articular cartilage in arthritis but in fact may affect cartilage indirectly [37]. Furthermore, in bovine menisci, the gene expression of *ADAMTS4* is preferentially upregulated by IL-1α in inner meniscal zones, whereas the gene expression of *ADAMTS5* is preferentially upregulated by TNF-α in outer meniscal zones [38]. In human OA synovium, upregulation of ADAMTS4, unlike that of ADAMTS5, was IL-1- and TNF-α-dependent, again exemplifying the fact that ADAMTS5 may be constitutively expressed and more active in joint structures other than articular cartilage [3]. The differential roles of the aggrecanases may add complexity to potential treatments discussed below. However, evidence of their cooperative roles in cartilage degradation and common activation by inflammatory cytokines suggests that both ADAMTS4 and ADAMTS5 represent important therapeutic targets in arthritis.

## Current arthritis treatments

Current strategies for arthritis treatment have favorable outcomes in patients who adequately respond. However, many treatment regimens are inadequate because of poor and often patient-specific efficacy; they also focus on decreasing pain and inflammation associated with the disease but often fail to effectively inhibit cartilage destruction and therefore the progression of the disease. Furthermore, because many treatments lose efficacy over time, the increasing doses that are often required augment their toxicity and side effects. Current efficacious treatments for RA predominately include disease-modifying anti-rheumatic drugs (DMARDs) or biological agents such as antibodies, and corticosteroid intra-articular joint injections and non-steroidal anti-inflammatory drugs (NSAIDs) are also used for both RA and OA.

### Rheumatoid arthritis

#### Disease-modifying anti-rheumatic drugs

**Figure 2:**
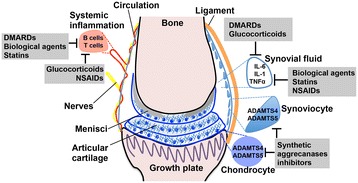
**The structure of the articular joint and targeted arthritis therapeutics.** Multifaceted components such as ligaments, menisci, and articular surfaces of the articular joint confer upon the joint compression-resistance and load-bearing properties. Arthritis may ensue if one or more components are compromised. Chondrocytes (dark blue) and synoviocytes (light blue) are a source of cytokine production and aggrecanase activity; inflammatory cytokines are found in synovial fluid of arthritic joints. Emerging therapeutics such as statins may suppress inflammatory cytokine activity in synovial fluid, thereby potentially inhibiting cartilage degradation mediated by ADAMTS4 and ADAMTS5. Disease-modifying anti-rheumatic drugs (DMARDs), biological agents, corticosteroids, and non-steroidal anti-inflammatory drugs (NSAIDs) may also prevent cartilage destruction indirectly by reducing inflammation. ADAMTS, A Disintegrin-like And Metalloproteinase domain with Thrombospondin-1 repeats; IL, interleukin; TNF-α, tumor necrosis factor-alpha.

DMARDs are essentially immunosuppressants used for the treatment of RA and include methotrexate, sulfasalazine, hydroxychloroquine, and leflunomide. Methotrexate is the ‘gold standard’ treatment and now a first-line therapy for RA patients early in the course of their disease, inhibiting purine and pyrimidine metabolism with its systemic anti-inflammatory effects (Figure 2) mediated through adenosine metabolism. Methotrexate suppresses proliferation of synovial fibroblasts, a source of aggrecanase production, and also modulates cytokine production [39]; therefore, methotrexate has the potential to slow cartilage destruction through the inhibition of ADAMTS4 and ADAMTS5 activity (Figure 1), although this possibility has yet to be investigated. Side effects of methotrexate include abdominal discomfort, alopecia, oral ulcerations, and cytopenia, which limit its use to low doses, compromising its efficacy (Table 1). Despite the improvements this treatment has offered, RA often persists after methotrexate regimens, and the multifactorial nature of RA means that some patients have a poor response to treatment regardless of the dose rate and time of administration. Despite the improvements this treatment has offered, the multifactorial nature of RA means that some patients remain unresponsive regardless of the dose rate and administration time. Therefore, additional DMARDs or biological agents are often administered in combination with methotrexate to improve disease outcomes.

**Table 1:** **Current and emerging arthritis treatments and their mode of action and side effects**

**Therapeutic agent**	**Mode of action**	**Side effects**
DMARDs		
Methotrexate [39]	Inhibits purine and pyrimidine synthesis and suppresses cytokine synthesis	Abdominal discomfort, alopecia, oral ulcerations and cytopenia, diarrhea, nausea, alopecia, vomiting, hepatotoxicity, and infections
Chloroquine, quinachrine, and hydroxychloroquine [40]	Anti-inflammatory, inhibition of cytokine diffusion	
Sulfasalazine [41]	Decreases pain and swelling	
Leflunomide [42]	Inhibits *de novo* pyrimidine synthesis	
Biological agents		
Infliximab [47,54]	TNF inhibitors	Nausea, upper respiratory tract infections, dyspepsia, and headaches
Golimumab [44]		
Adalimumab [46]		
Etanercept [45]		
Rituximab [50]	B-cell inhibitor (CD20)	
Abatacept	T-cell inhibitor	
Tocilizumab [43,48]	IL-6R inhibitor	
Anakinra	IL-1R inhibitor	
Corticosteroids/NSAIDs		
Prednisolone [54]	Anti-inflammatory	Weight gain, immunosuppression, altered glycemic control, glaucoma, hypertension, and osteoporosis
Aspirin (acetylsalicylic acid)	Anti-inflammatory	Gastrointestinal ulcer perforation and bleeding, renal impairment, and platelet dysfunction
Ibuprofen	Anti-inflammatory	
Naproxen	Anti-inflammatory	
Therapeutic agent	Mode of action	Key observations
Statins		
Simvastatin [56-58,66]	Anti-inflammatory	Suppression of macrophage infiltration and bone destruction (rat), decreased MMP-3 (rat), decreased migration and invasion of fibroblast-like synoviocytes (cells), and mild improvements in DAS28 scores and swollen joint counts (human)
Atorvastatin [59,60,64]	Anti-inflammatory Cartilage anabolism Cartilage protection	Decreased systemic TNF-α (human); reduction in C-reactive protein and erythrocyte sedimentation and improved DAS28 scores (human); and decreased IL-1β and MMP-13 and increased aggrecan and *ColIIa1* (*in vitro* human osteoarthritic chondrocytes)
Rosuvastatin [63]	Anti-inflammatory	Reduction of C-reactive protein (human)
Mevastatin [65]	Anti-inflammatory Cartilage protection	Reduction in IL-1β, MMP-3, and MMP-13 (rabbit)

Common arthritis treatments that target inflammation and pain are shown. Disease-modifying anti-rheumatic drugs (DMARDs) used for rheumatoid arthritis target systemic inflammation, whereas biological agents target localized inflammatory cytokines. Corticosteroids and non-steroidal anti-inflammatory drugs (NSAIDs) are often used for both rheumatoid arthritis and osteoarthritis treatments. Cholesterol-lowering drugs (statins) target inflammation and prevent cartilage breakdown in varying rheumatoid arthritis and osteoarthritis contexts. DAS28, Disease Activity Score in 28 Joints; IL, interleukin; MMP, matrix metalloproteinase; TNF, tumor necrosis factor.

Other DMARDs used to treat RA include the anti-malarial drugs chloroquine, quinachrine, and hydroxychloroquine. Although their mode of action is not well defined, they too are suppressors of inflammation (Figure 2) and thus are also useful therapies, especially in the case of systemic lupus erythematosus, in which quinachrine acts as a potent inhibitor of cytokine diffusion [40]. Sulfasalazine belongs to the ‘sulfa’ class of drugs, which includes a combination of salicylate (main ingredient of aspirin) and a sulfa antibiotic, and functions by decreasing pain and swelling to improve joint function [41]. Leflunomide is another DMARD whose effects are unsurprisingly comparable to those of methotrexate [42]; given that it is an immunomodulatory agent that also inhibits *de novo* pyrimidine synthesis, it also has the potential to indirectly inhibit ADAMTS4 and ADAMTS5 aggrecanase activity and cartilage destruction by reducing cytokine production (Figure 1). However, like methotrexate, these DMARDs have common and often unacceptable side effects such as diarrhea, nausea, alopecia, vomiting, hepatotoxicity, and infection (Table 1).

#### Biological agents

Biological agents used to treat arthritis comprise antibodies against inflammatory cytokines or their receptors to suppress their activity, and their use in RA is predominant. TNF-α inhibitors are the longest existing biological therapies and include adalimumab, entanercept, infliximab, and golimumab; they are the next line of defense either after or in combination with DMARD treatments. TNF-α inhibitor therapy precedes the discovery of the role of ADAMTS4 and ADAMTS5 in cartilage destruction; however, it is now apparent that inhibiting TNF-α may lead to a reduction in aggrecanase activity (Figure 1). Indeed, clinical trials demonstrated that TNF-α inhibitor administration in combination with methotrexate resulted in improvements in the symptoms of RA, including slowed radiographic disease progression in the majority of patients with active RA [43-45], an effect that could be attributable to reduced ADAMTS4 and ADAMTS5 aggrecanase activity. These inhibitors included etanercept, a human soluble and dimeric TNF type II receptor linked to an IgG_1_-Fc moiety that binds to and inactivates TNF-α [45], and golimumab, a humanized anti-TNF-α monoclonal antibody that has a high selectivity for human TNF-α, also effectively neutralizing its activity [44]. Other examples of efficacious TNF-α inhibitors include the human IgG_1_ monoclonal antibody adalimumab that binds specifically to TNF-α, preventing bioavailability to its p55 and p75 receptors [46], and infliximab, which is a chimeric (human-mouse) monoclonal antibody against human TNF and (like the above anti-TNF-α antibodies) has had success with symptomatic relief and improving quality of life in patients with RA [47]. However, despite the high efficacy of TNF-α inhibitors, approximately 30% of patients with RA have an unsatisfactory response [48], and side effects include nausea, upper respiratory tract infections, dyspepsia, and headaches (Table 1).

After failure of responsiveness to TNF-α inhibitors, other biological agents have been trialed with some success. These include rituximab (B-cell inhibitor), abatacept (T-cell inhibitor), tocilizumab (IL-6 inhibitor), and anakinra (IL-1 inhibitor) (Table 1). Treatment with tocilizumab, a human monoclonal anti-IL-6 receptor antibody that competitively inhibits the binding of IL-6 to its receptor [43,48], has demonstrated a significant reduction in symptoms of RA compared with DMARDs alone or in combination with methotrexate or other DMARDs [43]. Tocilizumab and anakinra are of particular interest to aggrecanase biology given that activities of ADAMTS4 and ADAMTS5 are regulated by IL-6 and IL-1 (Figure 1).

Abatacept, on the other hand, prevents T cells from recognizing antigen-presenting cells, as it comprises a fused Fc domain of IgG with human T-lymphocyte antigen 4. Abatacept has proven to be as effective as other biological agents in patients who failed to respond to TNF-α inhibitors. Emery [49] provided an excellent review of clinical data underlying those and previous trials of non-TNF-α inhibitor responders. Rituximab, a chimeric monoclonal antibody that leads to peripheral B-cell depletion by blocking the cell surface antigen CD20, demonstrated greater improvement in patients who had also failed to respond to anti-TNF-α therapy compared with placebo in a phase III study [50]. In combination with background methotrexate, rituximab treatment resulted in significant improvement in most disease scores; however, patients did suffer mild to moderate side effects, including low rates of infection.

As discussed above, IL-1, IL-6, and TNF-α are all regulators of ADAMTS4 and ADAMTS5 levels and activity and thus indirectly stimulate cartilage destruction. Therefore, any biological agent that targets systemic or local mediators of inflammation has the potential to inhibit cartilage destruction through regulating the bioavailability or bioactivity (or both) of ADAMTS4 and ADAMTS5. In most of the cases of the biological agents outlined above, which are currently used in the clinic, slowed radiographic disease progression has been demonstrated, strongly suggesting that aggrecanase activity was concurrently suppressed.

### Osteoarthritis

#### Corticosteroids and non-steroidal anti-inflammatory drugs

Glucocorticoids such as prednisolone are steroidal-based drugs administered orally or by intra-articular injection and have immunomodulatory properties and potent systemic and local anti-inflammatory effects, offering another treatment option for both OA and RA (Figure 2). Their short-term use is often indicated in acute joint injuries, joint replacement surgery, and tendonitis to suppress inflammation [51-53]. It has been suggested that glucocorticoids, often used in combination with DMARDs in RA, may be just as powerful in combination with a DMARD as a biological agent such as infliximab [54]. Glucocorticoids are used to manage acute pain and inflammation as they inhibit nuclear factor-kappa-B (NF-κB), a potent mediator of cytokine signaling. Given their immunosuppressive properties, glucocorticoids are also likely to lead to the suppression of ADAMTS4 and ADAMTS5 aggrecanase activity (Figure 1). Their disease-modifying properties have become more apparent recently; they may slow the progression of the disease, even after halting treatment [54]. However, although glucocorticoids are often quite effective, their adverse effects are problematic and include weight gain, osteoporosis, immunosuppression, altered glycemic control, glaucoma, fractures, muscle wasting, and hypertension (Table 1), which are counter-balanced by using the lowest possible dose for the shortest period of time.

NSAIDs such as aspirin (acetylsalicylic acid), ibuprofen, naproxen, and mobic are recommended as the first line of treatment in inflammatory arthritis because of their pain- and stiffness-relieving properties but also are an effective treatment for OA. They inhibit cyclooxygenase (COX), and some are specific to COX-2, which catalyzes the synthesis of prostaglandins. Recent evidence demonstrates that celecoxib, a selective COX-2 inhibitor, can diminish cyclic tensile strain-induced upregulation of ADAMTS5 and increase aggrecan expression in porcine mandibular chondrocytes [55], suggesting that NSAIDs may provide dual inhibition of inflammation and cartilage destruction. However, there are concerns about their long-term safety and efficacy as they are associated with toxicity and their adverse effects are dose-dependent and include gastrointestinal ulcer perforation and bleeding, renal impairment, and platelet dysfunction (Table 1).

## Statin therapy: an emerging treatment for rheumatoid arthritis and osteoarthritis

Statins - 3-hydroxy-3-methylglutaryl coenzyme A reductase inhibitors, including atorvastatin, mevastatin, pravastatin, and simvastatin - have roles predominately in cholesterol reduction and are effective in reducing cardiovascular morbidity and mortality. However, they also appear to have pleiotropic actions independent of their cholesterol-lowering properties such as anti-inflammatory effects, as demonstrated in experimental models of arthritis as well as in human trials (Table 1).

### Rheumatoid arthritis

There is evidence to suggest that statins have strong anti-inflammatory effects in RA. Simvastatin decreased articular macrophage infiltration and suppressed bone destruction in an RA rat model [56]. Furthermore, simvastatin inhibited the migration and invasion of fibroblast-like synoviocytes by preventing the activation of RhoA, a small GTP-binding protein known to activate NF-κB, therefore identifying a novel therapeutic agent for RA [57]. A prospective study in patients with RA demonstrated that 20 mg/day of simvastatin was a safe treatment that had anti-inflammatory effects with mild clinical improvements in measures such as swollen joint counts and Disease Activity Score in 28 Joints (DAS28) scores [58]. Disease activity also improved in RA patients undergoing a methotrexate regimen in combination with atorvastatin; evidence that inflammatory cytokines such as TNF-α were decreased systemically provided a potential mechanism to explain these observations [59]. A different trial of atorvastatin in patients with RA revealed a clinically apparent improvement in DAS28 scores as well as a decrease in C-reactive protein and erythrocyte sedimentation, which are typical markers used to diagnose RA [60].

However, conflicting evidence regarding the effects of statins has also arisen. Statins accelerated the effect of collagen type II-induced arthritis in mice [61]. Furthermore, statins may induce a pro-inflammatory response in peripheral blood mononuclear cells by activating IL-18 and caspase-1 [62]. Although rosuvastatin has been shown to reduce C-reactive protein in patients with RA, this effect did not correlate with an improvement in overall RA disease activity [63]. Therefore, statins may have both anti- and pro-inflammatory effects, depending on the form and progression of the disease, the type of statin prescribed, and whether the patient is undergoing a multiple-drug regimen. Further investigation into the effects of statins is required in RA to clarify whether they are clinically effective anti-inflammatory treatments in human trials and whether a corresponding reduction in aggrecanase activity in RA is apparent.

### Osteoarthritis

In OA chondrocyte cultures, atorvastatin produced a significant reduction in IL-1β and MMP-13 as well as an increase in aggrecan and *ColIIa1* expression, and this is an indication that atorvastatin may have chondroprotective effects, as well as anti-inflammatory effects [64], which could be relevant in the treatment of OA. This was also demonstrated with mevastatin, which showed reduced inflammatory cell infiltration and IL-1β and matrix-degrading enzyme (MMP-3 and MMP-13) expression in a rabbit model of experimental OA [65]. Furthermore, in a rat model of mechanically induced knee OA, simvastatin produced anti-inflammatory and immunomodulatory effects via the inhibition of MMP-3, demonstrating a possible additional chondroprotective effect [66]. Therefore, several classes of statins not only may have anti-inflammatory effects but also may demonstrate chondroprotective effects in patients with OA or RA.

Further investigations regarding the effects of statins in patients with OA or RA are clearly required given the likelihood of high incidences of co-morbidities with hypercholesterolemia, cardiovascular disease, obesity, and arthritis. However, given their apparent effectiveness in reducing inflammation or cytokine activity or both, one might hypothesize that affected joints of co-morbid patients undergoing a statin regimen could be inadvertently protected from cartilage destruction (Figures 1 and 2) to varying extents.

## The development of small-molecule inhibitors of the aggrecanases ADAMTS4 and ADAMTS5

Although there are several treatment options of varying efficacy for arthritis, many alternatives are currently being explored, especially those that selectively inhibit some MMPs or, more specifically, the ADAMTS aggrecanases (Table 2). Synthetic broad-spectrum MMP inhibitors such as batimastat and marimastat showed promise as anti-cancer therapies in the 1990s as they reduced tumor growth and spread in various animal models [67-72]. However, after the advancement of these inhibitors into phase II clinical trials, these inhibitors caused severe toxicity and advanced disease progression in several studies [73-76]. This has led to a more targeted approach to developing MMP and ADAMTS inhibitors as therapeutic interventions in disease.

**Table 2:** **The development of synthetic ADAMTS aggrecanase inhibitors**

**Compound**	**Target selectivity**
Engineered N-TIMP-3 [82]	ADAMTS4, ADAMTS5
(2*R*)-*N* ^4^-hydroxy-2-(3-hydroxybenzyl)-*N* ^1^-[(1*S*,2*R*)-2-hydroxy-,3-dihydro-1*H*-inden-1-yl] butanediamides [83]	ADAMTS4, ADAMTS5
N-hydroxyformamides [85]	ADAMTS4, ADAMTS5
1,2,4-triazole-3-thiol scaffolds [86]	ADAMTS5 > ADAMTS4
*N*-((8-Hydroxy-5-substituted-quinolin-7-yl)(phenyl)methyl)-2-phenyloxy/amino-acetamides [87]	ADAMTS5 > ADAMTS4,
5-((1*H*-Pyrazol-4-yl)methylene)-2-thioxothiazolidin-4-one [88]	ADAMTS5 > ADAMTS4
4-(benzamido)-4-(1,3,4-oxadiazol-2-yl) butanoic acid [89]	ADAMTS4, ADAMTS5
1-sulfonylaminocyclopropanecarboxylates, N-substituted sulfonylamino-alkanecarboxylates [90]	ADAMTS5
1,3,5-triazine core [91]	ADAMTS5
CRB017 (antibody against ancillary domain) [92]	ADAMTS5
AGG-523 (Pfizer Inc.)	ADAMTS4, ADAMTS5

Engineered and synthetic compounds are being developed to obtain selectivity toward the inhibition of the aggrecanases ADAMTS4 and ADAMTS5 to prevent cartilage destruction in both rheumatoid arthritis and osteoarthritis. ADAMTS, A Disintegrin-like And Metalloproteinase domain with Thrombospondin-1 repeats; TIMP, tissue inhibitor of metalloproteinase.

### Tissue inhibitors of metalloproteinases

TIMPs are specific endogenous MMP and ADAMTS inhibitors and also are essential for homeostasis of the joint and proper matrix turnover as described above. There are four mammalian TIMPs, designated TIMP-1 through −4; TIMP-1 was discovered in 1985. TIMP-3, unlike the other TIMPs, has a broad profile of inhibition that includes ADAMTS4 and ADAMTS5. TIMP-3 acts as a tumor suppressor and inhibitor of angiogenesis, and *Timp-3* homozygous-null mice present with enhanced TNF signaling and serum IL-6 levels [77], indicating a key role for TIMP-3 in innate immunity. Furthermore, *Timp-3* knockout mice present with an increased inflammatory response to antigen-induced arthritis and increased aggrecan and collagen II degradation with age [78,79]. TIMP-3 may be a suitable therapeutic treatment for patients with arthritis to suppress not only innate inflammatory cytokines in arthritis but also ADAMTS4 and ADAMTS5. Their lack of selectivity precludes them as an adequate treatment option in their native form. However, truncated TIMP-3 (N-TIMP-3), lacking its C-terminal domain, is a potent endogenous inhibitor of ADAMTS4 and ADAMTS5 with inhibition also demonstrated toward MMP-1, MMP-2, and (to a lesser extent) MMP-3 [80]. Furthermore, the thrombospondin type-1 repeats of ADAMTS4 and ADAMTS5 promote binding with N-TIMP-3 [81], providing further evidence that N-TIMP-3 may effectively inhibit ADAMTS4 and ADAMTS5 with high affinity. Moreover, by engineering the reactive site through amino acid substitutions within the N-terminus of N-TIMP-3, some selectivity toward ADAMTS4 and ADAMTS5 has been gained without off-target MMP inhibition [82], suggesting that with further modifications TIMPs may be a powerful potential future therapeutic.

### Synthetic inhibitors of aggrecanases

The need to develop novel and selective aggrecanase inhibitors has become increasingly essential to arthritis research. A series of (2*R*)-*N*^4^-hydroxy-2-(3-hydroxybenzyl)-*N*^1^-[(1*S*,2*R*)-2-hydroxy-,3-dihydro-1*H*-inden-1-yl] butanediamide derivatives have previously been developed as potent and selective inhibitors of aggrecanase activity [83]. A 3-hydroxyl group on one of the inhibitors achieved selectivity through hydrogen bonding with a threonine in the S1 pocket of ADAMTS5; however, this threonine is conserved in ADAMTS4, reducing the selectivity of these derivatives. A valine residue replaces this threonine in active sites of most of the MMPs, thereby achieving some selectivity toward the aggrecanases [84]. Recently, a series of novel achiral N-hydroxyformamide inhibitors of ADAMTS4 and ADAMTS5, which are highly selective and potent *in vitro*, have emerged [85]. In addition, a new family of ADAMTS5 inhibitors such as non-hydroxamic inhibitors, which display a 1,2,4-triazole-3-thiol scaffold as a putative zinc-binding group, have reached a reasonable level of selectivity toward ADAMTS5 [86].

*N*-((8-Hydroxy-5-substituted-quinolin-7-yl)(phenyl)methyl)-2-phenyloxy/amino-acetamide inhibitors have been synthesized, and four of these inhibitors demonstrated greater ADAMTS5 potency and selectivity over ADAMTS4 and MMP-13 [87]. In addition, 5-((1*H*-Pyrazol-4-yl)methylene)-2-thioxothiazolidin-4-one inhibitors have been synthesized and have shown good selectivity of ADAMTS5 over ADAMTS4 [88]. Yet another series of compounds, α-glutamic acid scaffold based 4-(benzamido)-4-(1,3,4-oxadiazol-2-yl) butanoic acids, have been designed and synthesized to inhibit the activity of both ADAMTS4 and ADAMTS5 [89]. Furthermore, a series of 1-sulfonylaminocyclopropanecarboxylates and N-substituted sulfonylamino-alkanecarboxylates are potent ADAMTS5 inhibitors with good selectivity over MMPs such as MMP-1 [90]. More recently, potent and selective novel ADAMTS5 inhibitor scaffolds which lacked a zinc-binding motif and which contained a 1,3,5-triazine core were designed [91].

A recent study ameliorated OA progression in a spontaneous OA mouse model by using intra-articular injections of an anti-ADAMTS5 antibody (CRB0017), showing the first evidence of a biological agent (antibody-mediated targeting) used against ADAMTS5 to halt the progression of OA (Figures 1 and 2) and a proof of principle that inhibiting this enzyme might be a promising therapeutic [92]. The aggrecanase inhibitor AGG-523 (Wyeth Pharmaceuticals, now part of Pfizer Inc., New York, NY, USA), which inhibits both ADAMTS4 and ADAMTS5, has undergone clinical trials for the treatment of OA and may become a new OA drug in the near future. Although it has yet to be established which aggrecanase is predominant in human arthritis, ADAMTS4 inhibitors still may be beneficial, particularly if they inhibit ADAMTS5 concurrently (Figures 1 and 2 and Table 2).

## Conclusions

Optimization and individualization of treatment strategies for patients with arthritis are required, as current treatments continue to fail in efficacy and often lead to serious side effects in a significant number of patients with arthritis. Ensuring that arthritis sufferers have the best treatment regimens for their condition is paramount. First- and second-line treatments, alongside combination therapy, show promising advancements in the field. Further research into prospective treatments, as well as into the mechanisms underlying joint destruction, is imperative. Questions such as which ADAMTS aggrecanase may have the most significant role in arthritis remain unanswered; however, it is clear that current and emerging therapeutics that could indirectly or directly inhibit these enzymes often lead to favorable outcomes in arthritis sufferers. The plethora of emerging small-molecule ADAMTS aggrecanase inhibitors gives hope that some will be efficacious and tolerable and that those that are specific to ADAMTS5 could be developed as novel therapeutics for arthritis patients in the near future. Thus, ADAMTS aggrecanase inhibitors may well become useful as both isolated and combinational therapies for most types of arthritis.

## Abbreviations

ADAMTS: A Disintegrin-like And Metalloproteinase domain with Thrombospondin-1 repeats
COX: Cyclooxygenase
CS: Chondroitin sulphate
DAS28: Disease Activity Score in 28 Joints
DMARD: Disease-modifying anti-rheumatic drug
IGD: Interglobular domain
IL: Interleukin
MMP: Matrix metalloproteinase
NF-κB: Nuclear factor-kappa-B
NSAID: Non-steroidal anti-inflammatory drug
OA: Osteoarthritis
RA: Rheumatoid arthritis
TIMP: Tissue inhibitor of metalloproteinase
TNF-α: Tumor necrosis factor-alpha
